# Comparison of Opioids Prescribed for Patients at Risk for Opioid Misuse Before and After Publication of the Centers for Disease Control and Prevention’s Opioid Prescribing Guidelines

**DOI:** 10.1001/jamanetworkopen.2020.27481

**Published:** 2020-12-02

**Authors:** Jeffrey F. Scherrer, Jane Tucker, Joanne Salas, Zidong Zhang, Richard Grucza

**Affiliations:** 1 Department of Family and Community Medicine, Saint Louis University School of Medicine, St Louis, Missouri

## Abstract

**Importance:**

It is not known whether decreases in Schedule II (high abuse potential) vs Schedule IV (lower abuse potential) opioid prescriptions overall and among high-risk patients followed publication of the Centers for Disease Control and Prevention (CDC) opioid prescribing guideline on March 15, 2016.

**Objectives:**

To compare the odds of new Schedule II opioid (codeine, hydrocodone, oxycodone) prescriptions vs Schedule IV opioid (tramadol) prescriptions in the 18-month periods before and after the CDC guideline release to determine whether new prescriptions for Schedule II opioids decreased relative to new prescriptions for tramadol and to assess whether patients with benzodiazepine prescriptions or those with depression, anxiety, or substance use disorders had a greater decrease in receipt of Schedule II vs Schedule IV opioids.

**Design, Setting, and Participants:**

Cross-sectional study of Optum’s deidentified Integrated Claims-Clinical data set for 5 million US adults 18 months before and 18 months after March 15, 2016. Eligible patients were 18 years or older, free of HIV and cancer diagnoses, and had a noncancer painful condition. Patients received new prescriptions for codeine, hydrocodone, oxycodone, or tramadol. Data were analyzed from September 5, 2014, to September 14, 2017.

**Exposure:**

The CDC opioid prescribing guideline published on March 15, 2016.

**Main Outcomes and Measures:**

The odds of prescriptions for each Schedule II opioid vs tramadol after guideline publication.

**Results:**

Data from 279 435 patients were included in the study. The mean (SD) age of patients was 52.9 (16.5) years; 61% were female and 79.4% were White. The prevalence of new prescriptions for each drug before and after guideline publication was as follows: codeine, 7.1% vs 7.0%; hydrocodone, 47.4% vs 45.6%; oxycodone, 22.4% vs 24.0%; and tramadol, 23.0% vs 23.4%. Overall, the odds of being prescribed hydrocodone or oxycodone vs tramadol significantly decreased after guideline publication (odds ratios, 0.95; 95% CI, 0.91-0.98 and 0.86; 95% CI, 0.82-0.90, respectively). Odds of being prescribed a Schedule II opioid vs tramadol after vs before guideline publication were similar in patients with and without benzodiazepine comedication or psychiatric disorders.

**Conclusions and Relevance:**

In the 18 months after compared with the 18 months before publication of the CDC prescribing guideline, a 14% decrease in oxycodone prescriptions was observed relative to tramadol. Little change in prescriptions of other Schedule II opioids was observed. Schedule II opioids continue to be prescribed to high-risk patients 18 months after publication of the CDC guideline.

## Key Points

**Question:**

Have prescriptions for Schedule II opioids with high abuse potential, vs Schedule IV tramadol (lower abuse potential), decreased in the 18-month periods before and after publication of the Centers for Disease Control and Prevention opioid prescribing guideline?

**Findings:**

In this cross-sectional study of 279 435 patients with usable data, the odds of prescriptions for Schedule II opioids were similar before and after publication of the guideline with few exceptions.

**Meaning:**

This study’s findings suggest that, among patients at risk for opioid misuse, the odds of receiving a Schedule II opioid for noncancer pain vs tramadol in the 18-month periods before and after guideline publication were similar to the odds for those not at risk.

## Teaser

This cross-sectional study uses deidentified patient information from Optum’s deidentified Integrated Claims-Clinical data set to compare frequency of opioid prescriptions for patients at risk for opioid misuse in the 18-month periods before and after publication of the Centers for Disease Control and Prevention (CDC) opioid prescribing guideline.

## Introduction

The release of the Centers for Disease Control and Prevention (CDC) Guideline for Prescribing Opioids for Chronic Pain^1^ in March 2016 offered recommendations for opioid therapy in primary care patients with noncancer pain. The guideline’s publication was followed by accelerated declines in opioid prescribing rates, high-dose prescribing, and benzodiazepine coprescribing.^2,3,4^ Research on the effect of the guidelines has yet to assess whether the type of opioid prescribed has changed. In addition, no findings that indicate whether patients with psychiatric disorders who are at risk for opioid misuse^5,6^ are less likely to receive opioids with high abuse potential after guideline publication are available. We limited analysis to codeine, hydrocodone, oxycodone, and tramadol because they are the opioids most frequently dispensed in the United States.^7,8,9,10^ These opioids were also selected because tramadol, a Schedule IV drug, has been associated with fewer adverse events than hydrocodone and oxycodone^10^ and has a lower abuse potential than Schedule II drugs (eg, codeine, hydrocodone, and oxycodone).^11^

Although the CDC guidelines do not include advice on prescribing specific opioids, we expected the declines in dose, duration, and benzodiazepine coprescribing to be accompanied by increased use of tramadol relative to Schedule II opioids. We compared the odds of new Schedule II opioid (codeine, hydrocodone, oxycodone) prescriptions with the odds of Schedule IV opioid (tramadol) prescriptions for 18 months before and 18 months after release of the CDC guideline to assess whether (1) new Schedule II prescriptions decreased more than Schedule IV prescriptions, (2) benzodiazepine coprescriptions were associated with a greater decrease in Schedule II vs Schedule IV opioid prescriptions, and (3) high-risk patients who had depression, anxiety, or substance use disorders had a greater decrease in receipt of Schedule II vs Schedule IV opioids.

## Methods

We used Optum’s deidentified Integrated Claims-Clinical data set (2010-2018) for 5 million patients (≥18 years) throughout the United States. We used this database because it contains more data on patient characteristics than medical claims data and includes information on outpatient and inpatient encounters, academic and nonacademic health care systems, and commercially insured, government insured, and uninsured patients. The study outcome was opioid prescribing measured 18 months before and 18 months after the study exposure: CDC guideline publication on March 15, 2016.^1^ Because data were deidentified, this study was considered to be non–human subjects research by the Saint Louis University Institutional Review Board and therefore deemed exempt from the need for institutional review board approval or patient informed consent. We followed the Strengthening the Reporting of Observational Studies in Epidemiology (STROBE) reporting guideline for cross-sectional studies.

Data were included if patients had a noncancer pain diagnosis, were free of cancer and HIV, and had been enrolled in the database in the 6-month period prior to a new Schedule IV or II opioid prescription. Data on patients who received new prescriptions for codeine, hydrocodone, oxycodone, a combination medication (eg, hydrocodone-acetaminophen), or tramadol were also included. A new prescription was defined as one that followed a period of 6 months or more without a prescription for any opioid, including methadone. Patients could enter the study anytime they met eligibility criteria. If any demographic variable for a patient was missing, data for that patient were excluded. Data for 70 patients in the 18-month period before guideline publication and 74 patients in the 18-month period after guideline publication were excluded because patients were not identified as male or female. The cohort creation is shown in the eFigure in the Supplement. The total number of patients with usable data was 279 435 (141 219 before and 138 216 after guideline publication).

Detailed definitions for all variables are provided in the eTable in the Supplement. Risk factors for opioid misuse were defined using *International Classification of Diseases, Ninth Revision*, and *International Statistical Classification of Diseases and Related Health Problems, Tenth Revision*, coding algorithms for depression, anxiety disorders, alcohol and drug abuse or dependence, and nicotine dependence. Benzodiazepine coprescription was measured any time in the period before or after guideline publication.

Covariates included age, sex, race and ethnicity, clinician type at time of new opioid prescription, patient’s census geographic region, obesity, and painful conditions. We adjusted for chronological time at occurrence of a new opioid prescription. This was expressed as months since September 14, 2014 (first possible new opioid prescription date). Diagnostic codes for more than 900 conditions for which an opioid may be prescribed^12^ were used to categorize painful conditions such as arthritis, musculoskeletal pain, back pain, neuropathic pain, and headache pain.^13^ Covariates for the cohort before guideline publication were measured from September 14, 2014, to March 14, 2016, and covariates for the cohort after guideline publication were measured from March 15, 2016, to September 15, 2017.

### Statistical Analysis

The data analysis was performed from May to July 2020. Crude, bivariate associations of opioid type and covariates were compared for the 18-month periods before and after guideline publication using χ^2^ tests and independent samples *t* tests, with standardized mean difference measuring effect size. Odds of being prescribed a Schedule II opioid vs a Schedule IV opioid in the 18-month periods before and after guideline publication were compared in a fully adjusted multinomial logistic regression model, which included all covariates and risk factors for opioid misuse. Effect modification was tested by examining the interaction term of the variable before and after guideline publication and the effect modifier before and after guideline publication. Because effect modification analysis included 24 separate tests and comparisons, the false discovery rate–adjusted *P* value (q value) was used to adjust for multiple comparisons and test for significance.^14^ The significance threshold was *P* < .05, and all tests were 2-sided.

## Results

Data were obtained from 279 435 patients with a mean (SD) age of 52.9 (16.5) years; 61% were female and 79.4% were White. The prevalence of new prescriptions for each drug before and after guideline publication was as follows: codeine, 7.1% vs 7.0%; hydrocodone, 47.4% vs 45.6%; oxycodone, 22.4% vs 24.0%; and tramadol, 23.0% vs 23.4% (Table 1). The distributions of demographic factors, geographic region, obesity, benzodiazepine coprescriptions, diagnoses of psychiatric disorders, and clinician specialties were not meaningfully different (standardized mean difference, <10) in the 18-month period before vs the 18-month period after guideline publication.

**Table 1.** Type of Opioid Prescribed and Characteristics of Patients 18 Months Before and 18 Months After CDC Guideline Publication on March 15, 2016

	Total (N = 279 435), No. (%)	Before guideline publication (n = 141 219), No. (%)	After guideline publication (n = 138 216), No. (%)	SMD, %
Type of new opioid prescription				
Codeine	19 786 (7.1)	10 079 (7.1)	9707 (7.0)	−0.4
Hydrocodone	129 943 (46.5)	66 983 (47.4)	62 960 (45.6)	−3.8
Oxycodone	64 865 (23.2)	31 658 (22.4)	33 207 (24.0)	3.8
Tramadol	64 841 (23.2)	32 499 (23.0)	32 342 (23.4)	0.9
Demographic characteristics				
Age, mean (SD), y	52.9 (16.5)	52.2 (16.5)	53.5 (16.4)	7.6
Sex				
Female	170 406 (61.0)	86 460 (61.2)	83 946 (60.7)	−1.0
Male	109 029 (39.0)	54 759 (38.8)	54 270 (39.3)	1.0
Race				
African American	38 903 (13.9)	20 068 (14.2)	18 835 (13.6)	−1.7
White	221 742 (79.4)	111 766 (79.1)	109 976 (79.6)	1.0
Other or unknown^a^	18 790 (6.7)	9385 (6.7)	9405 (6.8)	0.6
Ethnicity				
Hispanic	14 890 (5.3)	7377 (5.2)	7513 (5.4)	0.9
Not Hispanic	250 569 (89.7)	126 945 (89.9)	123 624 (89.4)	−1.5
Unknown	13 976 (5.0)	6897 (4.9)	7079 (5.1)	1.1
Region				
Midwest	139 114 (49.8)	69 561 (49.3)	69 553 (50.3)	2.1
Northeast	22 542 (8.1)	10 676 (7.6)	11 866 (8.6)	3.8
South	85 678 (30.7)	44 344 (31.4)	41 334 (29.9)	−3.2
West	21 209 (7.6)	11 080 (7.9)	10 129 (7.3)	−2.0
Unknown	10 892 (3.9)	5558 (3.9)	5334 (3.9)	−0.4
Pain diagnosis				
Arthritis	171 208 (61.3)	82 288 (58.3)	88 920 (64.3)	12.5
Musculoskeletal pain	168 291 (60.2)	82 811 (58.6)	85 480 (61.9)	6.6
Back pain	137 777 (49.3)	70 059 (49.6)	67 718 (49.0)	−1.2
Neuropathic pain	31 744 (11.4)	16 244 (11.5)	15 500 (11.2)	−0.9
Headache pain	54 968 (19.7)	28 136 (19.9)	26 832 (19.4)	−1.3
Obesity	151 462 (54.2)	75 490 (53.5)	75 972 (55.0)	3.0
Benzodiazepine coprescription	49 169 (17.6)	25 462 (18.0)	23 707 (17.2)	−2.3
Depression	41 177 (14.7)	19 963 (14.1)	21 214 (15.4)	3.4
Anxiety disorders	43 196 (15.5)	20 610 (14.6)	22 586 (16.3)	4.8
Alcohol abuse or dependence	9216 (3.3)	4538 (3.2)	4678 (3.4)	1.0
Drug abuse or dependence	11 757 (4.2)	5619 (4.0)	6138 (4.4)	2.3
Smoking/nicotine dependence	90 818 (32.5)	42 862 (30.4)	47 956 (34.7)	9.3
Clinician type associated with prescription				
Anesthesiology/pain medicine	2272 (0.8)	1111 (0.8)	1161 (0.8)	0.6
Surgical specialty	46 420 (16.6)	22 667 (16.1)	23 753 (17.2)	3.0
Emergency/urgent care	50 991 (18.2)	26 682 (18.9)	24 309 (17.6)	−3.4
Hospital	2531 (0.9)	1299 (0.9)	1232 (0.9)	−0.3
Primary care	45 401 (16.2)	24 413 (17.3)	20 988 (15.2)	−5.7
Other specialty	99 146 (35.5)	49 514 (35.1)	49 632 (35.9)	1.8
Unknown	32 674 (11.7)	15 533 (11.0)	17 141 (12.4)	4.4

Abbreviations: CDC, Centers for Disease Control and Prevention; SMD, standardized mean difference.

^a^ Other races included Asian, Hispanic, and Native American.

As shown in Table 2, after adjusting for covariates, the odds of receiving hydrocodone and oxycodone after guideline publication were significantly lower than those in the period before guideline publication (odds ratios [ORs], 0.95; 95% CI, 0.91-0.98 for hydrocodone and 0.86; 95% CI, 0.82-0.90 for oxycodone). Benzodiazepine coprescriptions were associated with greater odds of receiving a prescription for hydrocodone or oxycodone compared with tramadol (ORs, 1.07; 95% CI, 1.04-1.10 for hydrocodone and 1.19; 95% CI, 1.15-1.23 for oxycodone). Depression and anxiety disorders were significantly associated with greater odds of receiving a hydrocodone prescription (ORs, 1.09; 95% CI, 1.05-1.12 for depression and 1.07; 95% CI, 1.04-1.11 for an anxiety disorder) and an oxycodone prescription (ORs, 1.23; 95% CI, 1.18-1.27 for depression and 1.16; 95% CI, 1.11-1.20 for an anxiety disorder) compared with tramadol. Alcohol abuse or dependence was significantly associated with lower odds of receiving a prescription for hydrocodone vs tramadol (OR, 0.89; 95% CI, 0.84-0.94), and drug abuse or dependence was significantly associated with greater odds of receiving a prescription for codeine or oxycodone vs tramadol (ORs, 1.13; 95% CI, 1.03-1.23 for codeine and 1.40; 95% CI, 1.32-1.49 for oxycodone).

**Table 2.** Fully Adjusted Multinomial Model Measuring the Association Between Factors Associated With Opioid Misuse and Type of New Opioid Prescription Between the 18-Month Periods Before and After CDC Guideline Publication^a^

Factor	OR (95% CI)		
	Codeine vs tramadol	Hydrocodone vs tramadol	Oxycodone vs tramadol
Patient cohort			
Before guideline publication	1 [Reference]	1 [Reference]	1 [Reference]
After guideline publication	0.97 (0.91-1.03)	0.95 (0.91-0.98)	0.86 (0.82-0.90)
Benzodiazepine coprescription	0.99 (0.94-1.04)	1.07 (1.04-1.10)	1.19 (1.15-1.23)
Depression	1.00 (0.95-1.05)	1.09 (1.05-1.12)	1.23 (1.18-1.27)
Anxiety disorders	1.00 (0.95-1.06)	1.07 (1.04-1.11)	1.16 (1.11-1.20)
Alcohol abuse/dependence	1.01 (0.92-1.12)	0.89 (0.84-0.94)	0.93 (0.87-0.99)
Drug abuse/dependence	1.13 (1.03-1.23)	0.99 (0.93-1.04)	1.40 (1.32-1.49)
Nicotine dependence	0.92 (0.88-0.95)	1.20 (1.18-1.23)	1.35 (1.31-1.38)

Abbreviations: CDC, Centers for Disease Control and Prevention; OR, odds ratio.

^a^ Adjusted for age, sex, race, ethnicity, pain diagnoses, obesity, clinician type and US geographic region, and chronological time of first eligible opioid prescription (months since first possible new opioid date of September 14, 2014).

As shown in Table 3, the odds of receiving a prescription for codeine compared with a prescription for tramadol in the 18-month period after vs the 18-month period before guideline publication were higher among those with benzodiazepine coprescriptions (OR, 1.21; 95% CI, 1.03-1.42) vs those without benzodiazepine coprescriptions (OR, 0.93; 95% CI, 0.87-0.99); however, false discovery rate–adjusted *P* values showed that this difference was not statistically significant (q = .07). Results also showed that the odds of receiving a Schedule II opioid prescription vs tramadol in the 18-month period after vs the 18-month period before guideline publication did not significantly differ by depression, anxiety disorders, drug or alcohol abuse or dependence, and nicotine dependence.

**Table 3.** Stratified Estimates of Type of New Prescription Opioid in the 18-Month Periods After vs Before CDC Guideline Publication According to Factors Associated With Opioid Misuse

Factor	OR (95% CI)			Overall interaction *P* value (FDR q value)^a^
	Codeine vs tramadol	Hydrocodone vs tramadol	Oxycodone vs tramadol	
Benzodiazepine				
No benzodiazepine (after vs before)	0.93 (0.87-0.99)	0.93 (0.89-0.97)	0.86 (0.82-0.90)	.02 (.17)
Benzodiazepine (after vs before)	1.21 (1.03-1.42)	1.02 (0.93-1.12)	0.88 (0.79-0.98)	
*P* value (FDR q value) comparing stratum ORs^a^	.004 (.07)	.09 (.40)	.74 (.74)	
Depression				
No depression (after vs before)	0.98 (0.91-1.05)	0.94 (0.90-0.98)	0.87 (0.83-0.91)	.08 (.40)
Depression (after vs before)	0.92 (0.76-1.10)	1.01 (0.91-1.13)	0.82 (0.73-0.93)	
*P* value (FDR q value) comparing stratum ORs^a^	.52 (.60)	.19 (.56)	.42 (.57)	
Anxiety				
No anxiety (after vs before)	0.97 (0.90-1.04)	0.93 (0.90-0.97)	0.86 (0.81-0.90)	.39 (.57)
Anxiety (after vs before)	0.97 (0.82-1.16)	1.02 (0.92-1.14)	0.90 (0.80-1.01)	
*P* value (FDR q value) comparing stratum ORs^a^	.96 (.96)	.11 (.40)	.43 (.57)	
Alcohol abuse/dependence				
No alcohol (after vs before)	0.97 (0.91-1.04)	0.94 (0.91-0.98)	0.86 (0.82-0.90)	.90 (.90)
Alcohol (after vs before)	0.94 (0.64-1.38)	0.98 (0.78-1.23)	0.83 (0.65-1.07)	
*P* value (FDR q value) comparing stratum ORs^a^	.87 (.87)	.73 (.73)	.78 (.78)	
Drug abuse/dependence				
No drug (after vs before)	0.98 (0.91-1.04)	0.94 (0.91-0.98)	0.87 (0.83-0.91)	.24 (.56)
Drug (after vs before)	0.81 (0.57-1.15)	1.00 (0.81-1.24)	0.78 (0.63-0.98)	
*P* value (FDR q value) comparing stratum ORs^a^	.30 (.57)	.57 (.63)	.39 (.57)	
Nicotine dependence				
No nicotine (after vs before)	0.95 (0.88-1.02)	0.94 (0.89-0.98)	0.85 (0.81-0.90)	.67 (.68)
Nicotine (after vs before)	1.04 (0.91-1.18)	0.97 (0.90-1.04)	0.89 (0.82-0.96)	
*P* value (FDR q value) comparing stratum ORs^a^	.24 (.56)	.42 (.57)	.49 (.60)	

Abbreviations: CDC, Centers for Disease Control and Prevention; FDR, false discovery rate; OR, odds ratio.

^a^ The FDR-adjusted *P* values (ie, the q values) were adjusted for age, sex, race, ethnicity, pain diagnoses, obesity, clinician type and US geographic region, and chronological time of first eligible opioid prescription (months since first possible new opioid date of September 14, 2014) before and after CDC guideline publication.

A sensitivity analysis was conducted to exclude patients who received medication-assisted therapy with buprenorphine in the 6-month period prior to a new Schedule IV or II opioid prescription. Data for a total of 382 patients were excluded because they received buprenorphine treatment: 169 before guideline publication and 213 after guideline publication. Results were nearly identical to the original results.

## Discussion

Except for a 14% decrease in the odds of patients receiving oxycodone vs tramadol, we found no evidence of substantial changes in the odds of receiving a prescription for a Schedule II opioid vs tramadol in the 18 months after, compared with the 18 months before, publication of the CDC opioid prescribing guideline. Unexpectedly, a larger decrease in prescriptions for hydrocodone and oxycodone vs tramadol was not observed among patients who already had benzodiazepine prescriptions or those with depression, anxiety disorders, and substance use disorders compared with patients without these risk factors.

Analysis of US pharmacy data revealed a 25.3% decrease in high-dose prescriptions and a drop of 16.9% in the opioid prescribing rate between 2015 and 2017.^3^ Hydrocodone is by far the most common opioid prescribed in the United States. Thus, our observation of a small (5%) decrease in hydrocodone prescriptions may partly explain the previously reported overall decline in opioid prescribing between 2015 and 2017. However, studies indicating a decline in opioid prescribing have not been limited to new prescriptions and are not directly comparable to the results reported in this study.^2,3^

### Limitations

Limitations include lack of data on dispensed prescriptions. We are unable to assess whether prescriptions were appropriate to patients’ pain severity and pain-related interference with daily life or functioning. Our observation period after publication of the CDC guideline was 18 months, which may be insufficient to detect longer-term trends. Results may not be generalizable to all health care systems, such as the Veterans Health Administration or those in other countries.

## Conclusions

Except for the 14% decrease in prescriptions for oxycodone relative to tramadol, our results suggest the guideline release was not associated with changes in the prescribing of Schedule II opioids vs tramadol. The odds of receiving a prescription for hydrocodone or oxycodone vs tramadol in the 18-month periods before and after guideline publication for patients with psychiatric disorders did not differ significantly from the odds for patients without psychiatric disorders. Continued education is warranted to reduce prescribing of opioids with high abuse potential to patients who already have prescriptions for benzodiazepines and those with comorbid psychiatric and substance use disorders.

## Notes

Supplement. **eTable.** Variable Definitions Table **eFigure.** Cohort Creation
