# Supplementary material for: Comparison of Opioids Prescribed for Patients at Risk for Opioid Misuse Before and After Publication of the Centers for Disease Control and Prevention’s Opioid Prescribing Guidelines
Source: JAMA Netw Open. 2020 Dec 2;3(12):e2027481. doi: 10.1001/jamanetworkopen.2020.27481 (PMC7711316; doi:10.1001/jamanetworkopen.2020.27481)
Supplement: Supplement. — eTable. Variable Definitions Table eFigure. Cohort Creation [file jamanetwopen-e2027481-s001.pdf]

Supplementary Online Content

Scherrer JF, Tucker J, Salas J, Zhang Z, Grucza R. Comparison of opioids prescribed for patients at risk for opioid misuse before and after publication of the Centers for Disease Control and Prevention's opioid prescribing guidelines. *JAMA Netw Open*. 2020;3(12):e2027481. doi:10.1001/jamanetworkopen.2020.27481

eTable. Variable Definitions Table

eFigure. Cohort Creation

This supplementary material has been provided by the authors to give readers additional information about their work.

© 2020 Scherrer JF et al. *JAMA Network Open*

**eTable. Variable Definitions Table**
Pre/Post cohorts –<br>Pre cohort – new opioid prescription for codeine, hydrocodone, oxycodone or tramadol 9/14/14 to 3/14/16<br>Post cohort – new opioid prescription for codeine, hydrocodone, oxycodone or tramadol 3/15/16 to 9/15/17
New opioid prescription – new prescription for codeine, hydrocodone, oxycodone or tramadol. New prescription is one where opioid free for 6 months prior.
Arthritis - ≥1 diagnosis:<br>ICD9 = 710.0, 710.1, 710.2, 710.3, 710.4, 710.8, 710.9, 711.x, 713.x-717.x, 718.0x, 718.1x, 718.2x, 718.3x, 718.5x, 718.6x, 718.7x, 718.8x, 718.9x, 719.x, 720.0, V13.4<br>ICD10 = M00.x to M02.x, M05.x, M06.x, M08.x, M12.x to M19.x, M23.x, M24.0x to M24.4x, M24.6x to M24.9, M25.x, M32.10, M33.20, M33.90, M34.0, M34.1, M34.9, M35.00, M35.01, M35.5, M35.9, M36.2, M36.3, M36.4, M43.4, M43.5x, M45.9, M79.6x, R26.2, R29.4, R29.898, Z87.39
Musculoskeletal pain - ≥1 diagnosis:<br>ICD9 = 725.x, 726.0, 726.1x, 726.2, 726.3x, 726.4, 726.5, 726.6x, 726.71, 726.72, 726.90, 727.00, 727.03, 727.04, 727.05, 727.06, 727.09, 727.2, 727.3, 727.49, 727.50, 727.51, 727.6x, 727.89, 727.9, 729.0, 729.1, 729.4, 729.5, 729.7x, 729.89, 729.9, 729.91, 729.92, 781.99, 830.x-848.x, 905.6, 905.7, V43.6x, V43.7. V48.3, V49.6x, V49.7x<br>ICD10 = M35.3, M60.x to M79.x, R29.898, R29.91, S03.x, S13.x, S16.x, S23.x, S33.x, S39.0x, S39.9x, S43.x, S46.x, S53.x, S56.x, S63.x, S66.x, S73.x, S76.x, S83.x, S86.x, S93.x, S96.x, Z96.6x, Z97.1x, Z89.x
Back pain - ≥1 diagnosis:<br>ICD9 = 720.1, 720.2, 720.8x, 720.9, 721.x-722.x, 723.0-723.3, 723.5-723.7, 723.9, 724.x, 756.1x<br>ICD10 = M43.2x, M43.6, M43.8x, M43.9, M46.0x, M46.1, M46.4x, M46.8x, M46.9x, M47.x, M48.0x, M48.1x, M48.2x, M48.3x, M48.8x, M48.9, M49.8x, M50.x to M51.x, M53.x to M54.x, M96.1, Q76.0 to Q76.3, Q76.4x, Q76.6
Neuropathy - ≥1 diagnosis:<br>ICD9 = 053.13, 072.72, 337.0x, 337.1, 353.x-357.x , 377.33, 377.34, 377.41<br>ICD10 = B02.23, B26.84, G90.0x, G99.0, G54.x to G65.x
Headache - ≥1 diagnosis:<br>ICD9 = 307.81, 339.x, 346.0x, 346.1x, 346.2x, 346.3x, 346.4x, 346.5x, 346.7x, 346.8x, 346.9x 784.0<br>ICD10 = G43.x, G44.x, R51
Obesity – BMI ≥30 or ICD code<br>ICD9 = 278.00, 278.01<br>ICD10 = E66.9, E66.01
Benzodiazepine - ≥1 prescription for alprazolam, chlordiazepoxide, clonazepam, clorazepate, diazepam, lorazepam, oxazepam

Depression - ≥2 outpatient diagnosis in a 12 month period or ≥1 inpatient diagnosis:<br>ICD9 = 296.2x, 296.3x, 311<br>ICD10 = F32.0-F32.5, F32.9<br>F33.0-F33.3, F33.4x, F33.9
Anxiety disorders - ≥2 outpatient diagnosis in a 12 month period or ≥1 inpatient diagnosis. Composite of PTSD, generalized anxiety disorder, panic disorder, obsessive compulsive disorder, social phobia or anxiety disorder unsepecified.<br>ICD9 = 309.81, 300.02, 300.01, 300.3, 300.23, 300.00<br>ICD10 = F43.1x, F41.1, F41.0, F42, F40.1x, F41.9
Alcohol abuse/dependence - ≥1 diagnosis:<br>ICD9 = 303.9x, 305.0x<br>ICD10 = F10.x
Drug abuse/dependence - ≥1 diagnosis.<br>ICD9 = 304.1x, 305.4x, 304.2x, 305.6x, 304.3x, 305.2x, 304.4x, 305.7x, 304.5x, 305.3x, 304.6x, 305.9x, 304.0x, 305.5x, 304.7x, 304.8x, 304.9x<br>ICD10 = F11.x – F16.x, F18.x, F19.x
Nicotine abuse/dependence - ≥ 1 diagnosis:<br>ICD9 = V15.82, 305.1<br>ICD10 = Z87.891, Z72.0, F17.20x, F17.21x
Provider type – Type of provider associated with new opioid prescription. Anesthesiology/pain (pain medicine, anesthesiology), Surgical specialty (ambulatory surgical center, bariatric, cardiovascular, colon and rectal, neurological, obstetrics/gynecology, oral and maxillofacial, orthopedic, plastic, surgery, surgery and surgical specialties, thoracic, transplant, trauma, urology, vascular), Emergency/Urgent Care (ambulance, emergency medicine, urgent care center), Hospital (birthing center, critical care medicine, general acute care hospital, hospital medicine, lithotripsy center, other non-acute hospital), Primary Care (family medicine, general practice, primary care), Other (all other specialties not listed otherwise), Unknown
Chronological time of new opioid prescription – Month since 9/14/14 that new opioid prescription for codeine, hydrocodone, oxycodone, tramadol occurred
Demographic information – Age at new prescription, gender (male vs female), race (African-American, Caucasian, other/unknown), Hispanic ethnicity (Hispanic, not Hispanic, unknown), Region – Census Bureau (Midwest, South, Northeast, Unknown)

© 2020 Scherrer JF et al. *JAMA Network Open*

eFigure. Cohort Creation

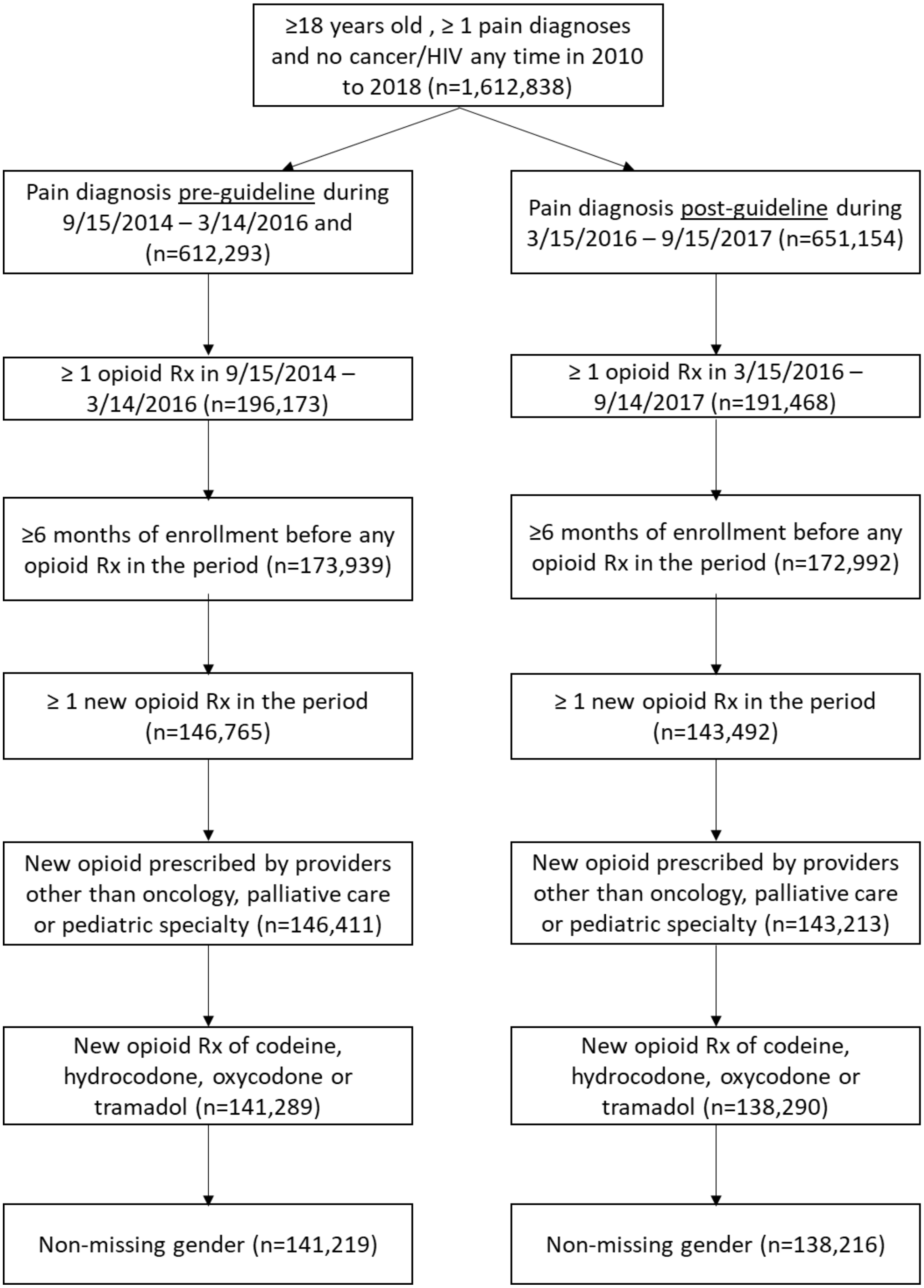

© 2020 Scherrer JF et al. *JAMA Network Open*
